# Seasonal influenza vaccine uptake among healthcare workers in tertiary care hospitals, Bangladesh: Study protocol for influenza vaccine supply and awareness intervention

**DOI:** 10.1186/s12889-022-14182-w

**Published:** 2022-09-24

**Authors:** Md Zakiul Hassan, Tahmina Shirin, Mahbubur Rahman, A. S. M. Alamgir, Nusrat Jahan, Md Abdullah Al Jubayer Biswas, Sazzad Hossain Khan, Md Ahmed Khairul Basher, Md Ariful Islam, Kamal Hussain, Md Nazrul Islam, Md Arif Rabbany, Md Azizul Haque, Shishir Ranjan Chakraborty, Syeda Rukhshana Parvin, Mahmudur Rahman, Fahmida Chowdhury

**Affiliations:** 1 Programme for Emerging Infections, Infectious Disease Division, International Centre for Diarrhoeal Disease Research, Bangladesh (icddr,b), Mohakhali, Dhaka, Bangladesh; 2 Nuffield Department of Medicine, University of Oxford, Oxford, UK; 3 Institute of Epidemiology, Disease Control and Research, Mohakhali, Dhaka, Bangladesh; 4 Department of Neonatology, Mymensingh Medical College, Mymensingh, Bangladesh; 5 Department of Medicine, Rajshahi Medical College, Rajshahi, Bangladesh; 6 Department of Medicine and Vice Principal, Sylhet MAG Osmani Medical College, Sylhet, Bangladesh; 7 Department of Paediatrics, Khulna Medical College, Khulna, Bangladesh; 8 Global Health Development (GHD), The Eastern Mediterranean Public Health Network (EMPHNET), Abdallah Ben Abbas St, Building No. 42, Amman, Jordan

**Keywords:** Influenza vaccination, Influenza vaccine awareness, Healthcare worker, Knowledge and perceptions, Barriers and motivators, Policymakers' views

## Abstract

**Background:**

Healthcare workers (HCWs), such as doctors, nurses, and support staffs involved in direct or indirect patient care, are at increased risk of influenza virus infections due to occupational exposures. Vaccination is the most effective way to prevent influenza. Despite the World Health Organization (WHO) recommendations, Bangladesh lacks a seasonal influenza vaccination policy for HCWs, and thus vaccination rates remain low. The current project aims to investigate the effect of interventions on influenza vaccine awareness and availability of vaccine supply, explore HCWs’ knowledge and perceptions about influenza vaccination, understand the barriers and motivators for influenza vaccine uptake, and understand policymakers' views on the practicality of influenza vaccination among HCWs.

**Method:**

We will conduct the study at four tertiary care teaching hospitals in Bangladesh, using a cluster randomized controlled trial approach, with the hospital as the unit of randomization and intervention. The study population will include all types of HCWs.The four different types of intervention will be randomly allocated and implemented in four study hospitals separately. The four interventions will be: i) ensuring the availability of influenza vaccine supply; ii) developing influenza vaccine awareness; iii) both ensuring influenza vaccine supply and developing influenza vaccine awareness and iv) control arm with no intervention. Both quantitative and qualitative approaches will be applied to assess the intervention effect. We will estimate the Difference in Differences (DID) with 95% CI of the proportion of vaccine uptake between each intervention and control (non-intervention) arm, adjusting for the clustering effect. The qualitative data will be summarised using a framework matrix method.

**Discussion:**

The results of this study will inform the development and implementation of a context-specific strategy to enhance influenza vaccination rates among Bangladeshi HCWs.

**Trial registration:**

Clinicaltrials.gov NCT05521763. Version 2.0 was registered in September 2022, and the first participant enrolled in March 2022. Retrospectively registered.

**Supplementary Information:**

The online version contains supplementary material available at 10.1186/s12889-022-14182-w.

## Background

Influenza annually affects a considerable fraction of the world's population, causing significant health, social, and economic consequences [1]. Globally, influenza infects approximately 5–15% of adults, and an estimated range of 291,000 to 646,000 persons die from seasonal influenza-related respiratory illnesses each year [2–4]. Influenza causes significant morbidity and mortality among healthcare workers (HCWs) due to occupational exposure [5–7]. After a comprehensive assessment of 26 research and a meta-analysis of 15 trials, Lietz et al. found that HCWs had an influenza prevalence of 6.3%, significantly higher than healthy working adults (5.1%) [7, 8]. During reported influenza outbreaks, the attack rate among HCWs ranged between 18% and 24%, higher than the general population (11%) [9, 10]. HCWs often work while experiencing influenza-like illness (ILI) (40–83%), increasing the likelihood of influenza transmission to colleagues and patients [6, 11].

Influenza vaccines are the most effective way to prevent infection and reduce the severity of the disease [12, 13]. Retrospective studies have shown correlations between increased influenza vaccination among HCWs and decreased nosocomial influenza in acute care settings [14–16]. Considering the disease burden of influenza, in 2012, the World Health Organization’s (WHO) Strategic Advisory Group of Experts (SAGE) on immunization recommended influenza vaccination for HCWs [17]. WHO SAGE declared HCWs as the highest priority risk group [18, 19].

Despite WHO vaccine recommendations and rising scientific data on influenza burden and awareness, vaccination coverage rates among HCWs in low- and middle-income countries (LMICs) continued to be low [18, 20, 21]. Moreover, less than half (46%; 64/138) of LMICs have national policies for seasonal influenza vaccination for HCWs [21]. Persistently low vaccination rates among HCWs in most LMICs raise international concerns about the broader spread of influenza to patients, particularly those at increased risk of influenza-like illness [1, 22, 23].

Studies have shown that interventions such as resources for education and training (e.g. posters, mass mailings, leaflets, newsletters, seminars, lectures, demonstrations, expert visits, and multimedia) enhance awareness and understanding of the influenza vaccine and improve vaccine uptake among HCWs [24]. Healthcare workers can also boost influenza vaccine uptake by offering incentives for vaccination, organizing campaigns to enhance vaccine knowledge, issuing vaccination reminders, and using lead vaccine advocates [24]. A cluster randomized controlled trial among healthcare workers of 33 Dutch nursing homes conducted in 2010 demonstrated that over the course of one influenza season, adopting education and institutional support increased vaccination uptake in the intervention group to 25%, whereas it was 16% in the control group [25].

Comprehensive reviews on the acceptance of the influenza vaccine by HCWs have identified several factors influencing vaccine uptake [26]. The primary motivations for HCWs’ influenza vaccination uptake were self-preservation and protecting family members avoiding sickness, or protecting patients [26]. At the same time, fear about vaccines’ safety, low-risk perception, refusal of the social benefit of the influenza shot, low social pressure, vaccination skepticism, not willingness to be flu-vaccinated, shortage of influenza-specific competence, and inaccessibility to get vaccination facilities were identified as a significant deterrent for vaccine uptake [26].

In Bangladesh, HCWs frequently reported hospital-acquired respiratory illnesses [27]. A study in Bangladeshi tertiary care hospitals found that 27% (61/226) of HCWs experienced a respiratory illness during the study period [27]. Each illness episode lasted an average of 4.3 days, resulting in a total of 27 days per HCW per year absent due to illness [27]. Moreover, with overcrowded hospitals and a lack of infection control practices, the nosocomial transmission of influenza would likely be amplified during a pandemic [28, 29]. This both escalates sickness absenteeism among the HCWs and reduces the workforce in the healthcare facilities [29].

There is currently no national influenza vaccination strategy in Bangladesh for the population at increased risk of influenza-like-illness such as HCWs, as recommended by WHO [30]. Each year, the Ministry of Health and Family Welfare (MoH&FW) of the People's Republic of Bangladesh provides free influenza vaccination to all Hajj pilgrims as a mandatory requirement imposed by the Saudi Health Ministry; this is the only group in Bangladesh that has a seasonal influenza vaccine policy [31]. Currently, only a few selected private vaccination centers in Dhaka offer influenza vaccines at a market-rated price (MRP). However, the number of doses administered per year by private vaccination centers remains unknown**.** Due to the limited availability of the vaccine in Bangladesh, the willingness to receive the influenza vaccine of risk group populations such as HCWs remains unclear.

To protect HCWs and reduce nosocomial influenza transmission in both epidemic and pandemic settings, the promotion of influenza vaccination for this risk group should be a priority. HCWs’ knowledge, attitudes, and perceptions about influenza and influenza vaccine influence their decision regarding vaccination. Understanding the barriers and motivation of vaccine uptake is required to tailor local influenza vaccination policies and implementation strategies to facilitate and improve vaccine uptake.

This study intends to determine the current status of influenza vaccination among HCWs and generate preliminary data on whether increased awareness and availability of influenza vaccine improve vaccine uptake and factors affecting the uptake rates. The critical data generated from the study would inform the development of an influenza vaccination policy for HCWs.

We hypothesize that increasing awareness and availability of influenza vaccine supply would increase influenza vaccine uptake among Bangladeshi HCWs. We formulated the research questions (RQ): What are the effects of influenza vaccination awareness or availability of vaccine supply or both in the study hospitals on HCWs' influenza vaccine uptake in Bangladesh?

## Methods

### Study setting

**Fig. 1:**
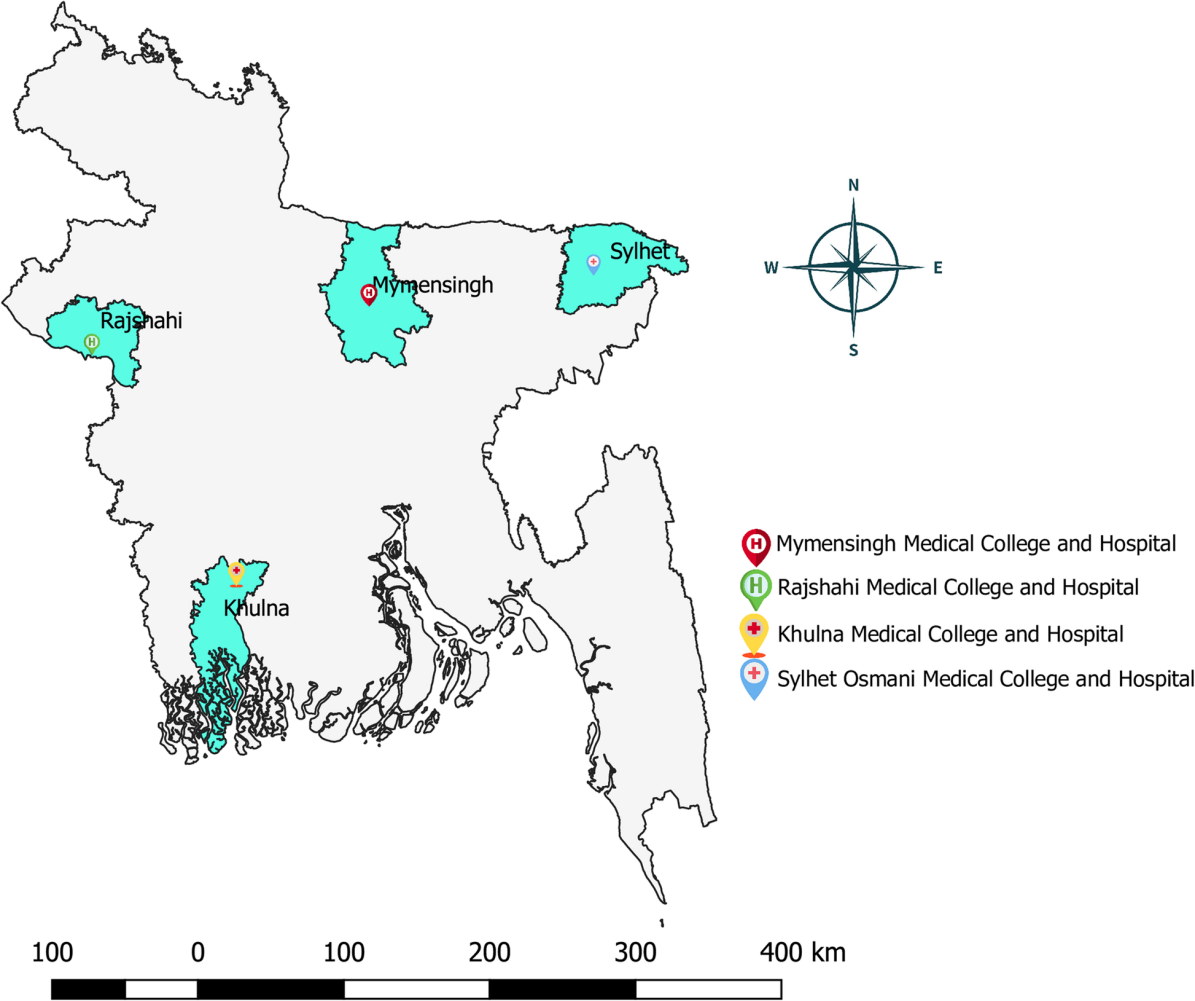
Location of the study sites. Source: Authors generated the map using QGIS version 3.2.1

The study will be conducted at four out of twenty-five conveniently selected tertiary-level public teaching hospitals in Bangladesh [32]. We have chosen the study hospitals considering several factors. Firstly, to minimize the diffusion of interventions, we have chosen hospitals from four different administrative divisions (i.e. geographically distant). Secondly, we have avoided hospitals where we have other ongoing studies among HCWs. Lastly, we have chosen tertiary-level public hospitals with a relatively higher number of HCWs to meet the minimum sample size requirement per study arm. The name and locations of the hospitals are as follows (Fig. 1):

1. Sylhet MAG Osmani Medical College Hospital, Sylhet, Bangladesh
2. Rajshahi Medical College Hospital, Rajshahi, Bangladesh
3. Khulna Medical College Hospital, Khulna, Bangladesh
4. Mymensingh Medical College Hospital, Mymensingh, Bangladesh

### Study design

**Fig. 2:**
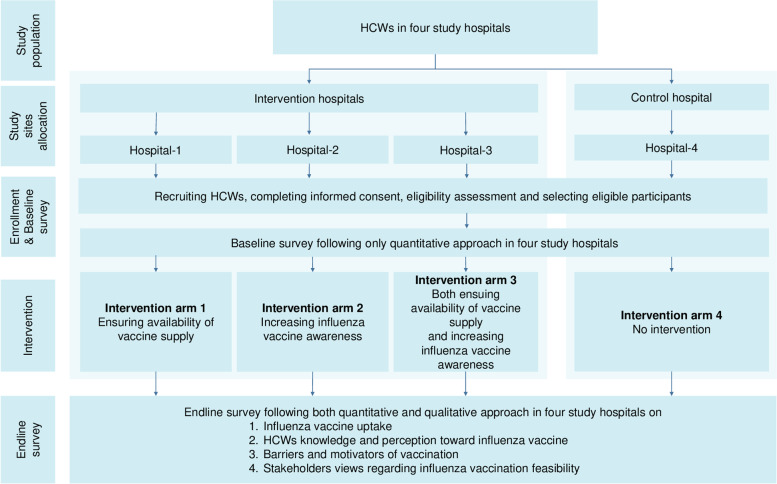
Schematic diagram of study method

**Fig. 3:**
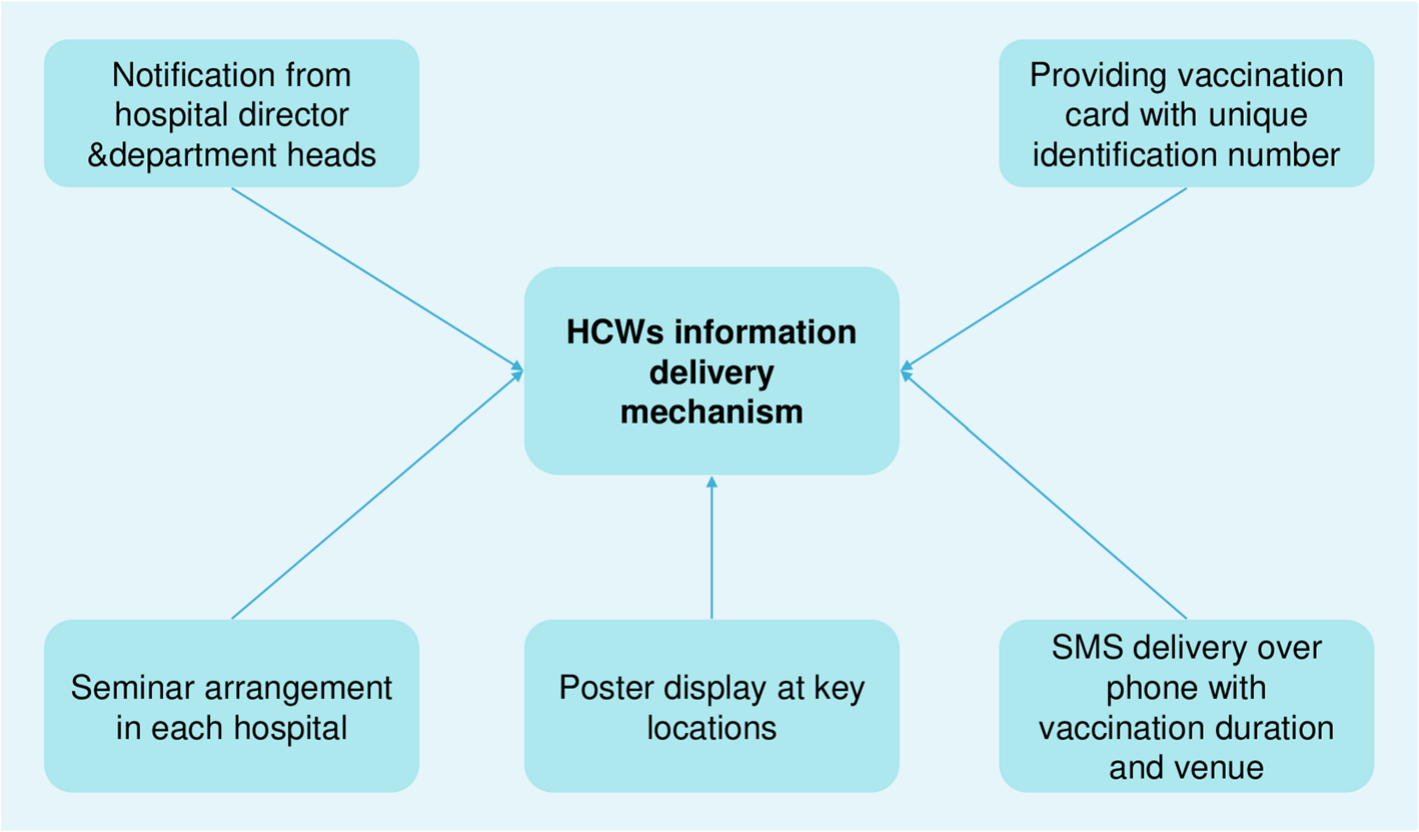
HCWs information delivery mechanism on influenza vaccination

We will use a cluster randomized controlled trial design with the hospital as the unit of randomization and intervention. The four different types of intervention will be randomly allocated and implemented in four study hospitals separately. The four interventions will be: 1^st^ intervention: ensuring availability of influenza vaccine supply; 2^nd^ intervention: developing influenza vaccine awareness activities; 3^rd^ intervention: both ensuring the availability of influenza vaccine supply and developing influenza vaccine awareness activities, and 4^th^ intervention: control arm with no intervention. We will assess influenza vaccine uptake before and after intervention and between different study arms. Moreover, barriers and motivators of vaccine uptake will be explored by applying both quantitative surveys and qualitative methods (i.e. in-depth interviews and focus group discussions) . We will also use a qualitative approach (i.e. key informant interviews) to understand the policymakers’ (such as hospital administration, influenza vaccine expert) perspectives and opinions on influenza vaccine uptake among HCWs. Figure 2 & Fig. 3 illustrates the study design and interventions.

### Study population and participant recruitment

Our study population will be all types of HCWs (Supplementary Table 1) involved in patient care working at the study facilities at the time the study begins; HCWs will be broadly categorized into doctors, nurses, and allied health personnel (supplementary table 1). HCWs who will not be directly or indirectly involved in patient care, such as basic medical science faculties (e.g., anatomy, physiology, biochemistry, forensic medicine, pathology, and microbiology), will not be included as participants. All eligible HCWs who will be working at the study hospitals during the implementation period and consent to participate in the study will be enrolled in the study as participants. We will collect the list of HCWs currently working in the facility from the hospital administration. Our team will approach the randomly selected HCWs and invite them to participate in the study. Eligible HCWs who will consent will be enrolled.

### Sample size and sampling

#### Quantitative survey

Our primary outcome of interest is influenza vaccine uptake among HCWs. An ongoing study among HCWs in Bangladesh at tertiary level facilities has shown a baseline influenza vaccine uptake of about 5% (Zakiul et al., personal communication). But comprehensive reviews on influenza vaccine uptake among HCWs have identified a wide variation of vaccine uptake rates globally between 13–53% [26]. We expect a similar uptake rate between 10–50% following awareness and availability of influenza vaccine supply in study hospitals. However, we assume an increase in the uptake rate of at least five percentage points for the sample size requirement.

For a one-sided test, given the allocation ratio R = n_2_/n_1_, the total sample size n is computed by the following formula:

$$n=\frac{{\left\{{z}_{1-\alpha \sqrt{\overline{p }\left(1-\overline{p }\right)}-{z}_{\beta }\sqrt{{w}_{2}{p}_{1}\left(1-{p}_{1}\right)+{w}_{1}{p}_{2}\left(1-{p}_{2}\right)}}\right\}}^{2}}{{w}_{1}{w}_{2}{\left({p}_{2}-{p}_{1}\right)}^{2}}$$

where uptake rate p_1_ = 5% in the control group and p_2_ = 10% in the intervention group, the pooled proportion $, \overline{p }$ = (n_1_p_1_ + n_2_p_2_)/(n_1_ + n_2_), and w_1_ = 1/(1 + R) and w_2_ = R/(1 + R). (Stata 13 manual; page 156). In our case, R = 1 for equal allocation, Z_1-α_ = 1.645 for 5% level of significance and Z_β_ = 0.84 for 80% power.

The estimated sample size is 435 HCWs in each arm. With a clustering (design) effect of 2 and a 10% non-response rate, a sample size of 957 HCWs is required for each arm. Consequently, the total sample size is 3,828.

#### Qualitative interviews

We will use qualitative methods to understand HCWs barriers and motivators for influenza vaccine uptake and policy makers’ perspectives on influenza vaccination feasibility. The qualitative methodologies will be in-depth interviews (IDIs) and focus group discussions (FGDs) among vaccinated and nonvaccinated HCWs. We will also conduct key informant interviews (KIIs) among policy makers.

For planning purposes, we anticipated a minimal sample size for each target group to reach saturation (Table 1 and Table 2), based on experiences from previous studies. This indicates a minimal sample size (Table 1 and Table 2). However, participants will be recruited until theoretical saturation for the research objective is reached, which is the point when no new insights are generated through the data.

**Table 1:** Sample size and sample distribution for in-depth interviews (IDI) and focus group discussions (FGD)

Type of interview method and selection criteria	Category	Target group	No. of the interview in each facility	Total no. of interviews in four hospitals
In-depth interviews (IDIs) participant selection (participants will be selected from years of experience, the domain of expertise and male: female ratio will be maintained)	Vaccinated	With doctors	1	4
		With nurses	1	4
		With allied health personnel	1	4
	*Nonvaccinated*	With doctors	1	4
		With nurses	1	4
		With allied health personnel	1	4
	**Total number of IDI participants**			**24**
Focus group discussions (heterogeneous in the combination of male–female participation; 8–10 participants in each discussion)	Vaccinated	With intern doctors		1
		With nurses		1
		With allied health personnel		1
	*Nonvaccinated*	With intern doctors		1
		With nurses		1
		With allied health personnel		1
	**Total number of FGDs**			**6**

**Table 2:** Sample size and sample distribution for key informant interviews (KIIs)

Target group	Number of KIIs
Director, Communicable Disease Control, Directorate General of Health Services(DGHS)	1
Director, IEDCR, DGHS	1
Line Director, EPI	1
Program manager, EPI	1
Deputy program manager, EPI	1
Key personnel from WHO	1
Current and retired influenza experts	2
Members of the National Immunization Technical Advisory Group (NITAG)	2
GAVI Bangladesh	1
Hospital directors	4
**Total number of interviews**	**15**

### Intervention

The intervention activities will be started before the influenza season, from May to September, in Bangladesh. Before the start of the influenza season, the study hospitals (4) will be randomly allocated into four study arms: (1) ensuring availability of influenza vaccine, (2) increasing influenza vaccination awareness, (3) ensuring the supply of influenza vaccine, and increasing awareness of influenza vaccine simultaneously (4) control arm with no intervention. We will take the following measures for specific arms of intervention hospitals to inform study participants about receiving influenza vaccination.

### Intervention arm-1: Intervention package with the availability of vaccine supply only

1.1. In Bangladesh, vaccine suppliers sell inactivated, quadrivalent influenza vaccines in both the Northern and Southern Hemisphere compositions of the vaccine [33]. The reason behind the availability of these vaccines is that Bangladesh is located in the tropical and subtropical southern and south-eastern Asia region, and country-specific data suggest that peak influenza activity occurs from May to September [34–36]. This vaccine contains influenza A(H3N2), pandemic A(H1N1) 2009 subtypes, and influenza B lineages B/Yamagata and B/Victoria. We will work with private suppliers to ensure vaccines are available at the hospital premises. Then a vaccination booth will be set up at the hospital premises, and administer the vaccine at the market-rated price (MRP). The booth will be open during the two-month intervention period and from 9 AM to 2 PM during each working day.

1.2. All HCWs will be notified about only vaccine availability information by the hospital director and respective department heads.

1.3. A list of all staff working in the study hospitals will be prepared, and they will be provided with an influenza vaccination record card with a unique identification number. The study staff will ensure receipt of vaccination cards for all participants.

1.4. A short message (SMS) will be delivered over the mobile phone number to all participants, e.g., vaccination program duration, venue, time, and vaccine price, to cascade vaccination information to participants.

1.5. Posters with only vaccine delivery information (i.e., vaccination duration, vaccination venue, vaccine price) will be displayed at key hospital locations, e.g., the main entrance of the hospital, nursing station, vaccine delivery point/place, doctors’ room, nurses’ room, intern doctors’ room, common room canteen, etc.

### Intervention arm-2: Intervention package with vaccination awareness only

2.1. The hospital director and respective heads will notify all HCWs about participating in the vaccination awareness program facilitated by the study team.

2.2. We will arrange separate seminars for each group of HCWs (doctors, nurses, and allied health personnel) in the hospital to sensitize participants about the significance of receiving influenza vaccines and the risk and benefits of influenza vaccination.

2.3. A list of all staff working in the study hospitals will be prepared, and they will be provided with an influenza vaccination record card with a unique identification number. The study staff will ensure receipt of vaccination cards for all participants.

2.4. A SMS will be delivered over the mobile phone number to all participants, e.g., time to vaccinate, vaccine price, and available places to purchase the vaccine.

2.5. Posters containing messages on the importance of influenza vaccination will be displayed at key hospital locations, e.g., the main entrance of the hospital, nursing station, vaccine delivery point/place, doctors’ room, nurses’ room, intern doctors’ room, common room canteen, etc. All the material will be adopted and contextualized from the available literature [37–39]. Figure 3 depicts the information delivery mechanism.

2.6. No vaccination booth will be set up at the hospital premises.

### Intervention arm-3: Intervention package with a combination of vaccine availability and awareness

3.1. We will work with the study hospital authority, influenza vaccine manufacturer, and supplier company to set up a vaccination booth at the hospital premises to administer the vaccine at the MRP by hospital nurses.

3.2. The hospital director and respective department heads will notify all HCWs to receive the offered influenza vaccine.

3.3. A list of all staff working in the study hospitals will be prepared, and they will be provided with an influenza vaccination record card with a unique identification number. The icddr,b, study staff will ensure receipt of vaccination cards for all participants.

3.4. We will arrange separate seminars for each group of HCWs (doctors, nurses, allied health personnel) in the hospital to sensitize participants about the objectives and implementation of the ongoing vaccination program as well as about the significance of receiving influenza vaccine and the risk and benefits of influenza vaccination.

3.5. A SMS will be delivered over the mobile phone number to all participants, e.g., vaccination program duration, venue, time, and vaccine price, to cascade vaccination information to optimum participants.

3.6. Posters with key messages will be displayed at crucial hospital locations, e.g., the hospital's main entrance, nursing station, vaccine delivery point/place, doctors’ room, nurses’ room, intern doctors’ room, common room canteen, etc.

### Control arm: No intervention

4.1. In the control facility, we will not intervene in the existing knowledge and practice about influenza vaccination of the HCWs.

### Implementing the vaccination program (for intervention arms 1 & 3)

A vaccination booth will be set up at the nurse’s station or in a common space in both hospitals. The vaccine will be provided at MRP among HCWs. The hospital nurses will administer the vaccine. After receiving the vaccine, HCWs will sign the vaccination card in front of the staff nurse to ensure receiving the vaccine receipt. During the two-month vaccination program, icddr,b staff will monitor the entire program and ensure vaccine and logistics availability in study hospitals.

To report any adverse events following immunization (AEFI), we will follow the existing surveillance channels established by the WHO and the MoHFW, the Government of Bangladesh. During the intervention briefing session at each study arm, we will provide information about reporting an AEFI (if any) within the hospital’s existing surveillance system as well as the hotline number (provided in the consent form) to inform such issue. Moreover, an icddr,b staff will be placed at each study hospital throughout the study implementation period. He/she will also monitor any AEFI issue and existing hospital surveillance setup. If any AEFI case is found, the icddr,b staff will immediately inform the Hospital Surveillance Officer (HSO), assist the HSO in filling in the AEFI report form, and submit it to the Expanded Programme on Immunization (EPI) headquarter through the Chief Health Officer. Besides, we will analyze the AEFI data (i.e., the number of AEFI reports received, number of AEFI by type, and classification of AEFI by cause) and share the findings with key stakeholders, including the Directorate General of Drug Administration (DGDA).

### Quantitative data collection

We will employ a quantitative method for evaluating the effect of the intervention. We will perform the following activities:

5.1. We will conduct a baseline survey among HCWs of the study hospitals to collect information on HCWs’ influenza vaccine uptake history and their knowledge and perception of influenza vaccination.

5.2 At the end of the influenza vaccine season, we will do a post-intervention survey at study facilities to collect similar information.

Trained staff will approach the participants to solicit their interest and consent. The staff will be well-spoken and discuss with the study participants regarding confidentiality, objectives of the survey, use of data, and utility of results. Those who agree to participate will be interviewed according to the survey questionnaire. The structured questionnaire will be developed and contextualized following a review of the literature from prior research [40–44]. The questionnaire will be focused on the number of vaccinated HCWs (evidence from the signed vaccination card), socio-demographic information of the participants, including age, gender, profession (doctor/nurse), and department; perception of influenza risk; perception of vaccine effectiveness and side effects, practices of recommending vaccination to family and patients, and practice and attitude towards a mandatory vaccination and their willingness to pay for future vaccination.

### Qualitative data collection

At the end of the intervention, qualitative interviews will be conducted. IDIs and FGDs will be used to understand vaccinated and nonvaccinated participants’ perceptions about influenza risk, vaccine effectiveness, side effects, and adverse effects. IDIs will be performed with both vaccinated and non-vaccinated HCWs to reveal an in-depth understanding of the factors and decisions for participating in vaccination. In addition, we will conduct FGDs with intern doctors (to understand group behavior and peer pressure on vaccination), nurses, and allied health personnel for data triangulation and comprehend the influence of group behavior on vaccine uptake.

To explore the feasibility of the influenza vaccination of HCWs, we will collect qualitative data through KIIs with purposively selected personnel and stakeholders. The qualitative data will be on experiences with influenza vaccination programs; barriers faced in implementing the vaccination program; existing workload; availability of infrastructural supports, logistics, and human resources; adequacy of cold chain and vaccine storage management; suitability of vaccination session management; and assessment of previous vaccination programs among health workers (if any), with particular attention to the feasibility of influenza vaccination among HCWs. We will summarise the interview data to prepare a policy brief and share it with the National Immunization Technical Advisory Group (NITAG).

### Data analysis

#### Quantitative data

We will conduct descriptive analysis to summarise the categorical variables using frequency, percentage, and 95% confidence interval (CI). We will summarise numerical or continuous variables using mean and standard deviation (SD) for symmetric distribution and median and interquartile range (IQR) for asymmetric distribution. We will estimate the Difference in Differences (DID) with 95% CI of the proportion of vaccine uptake between each intervention and control (non-intervention) arm, adjusting for the clustering effect and testing whether it is statistically significant or not at a 5% level of significance. We will also look for association and estimate the odds ratio (OR)/risk ratio (RR) with 95%CI between influenza vaccine uptake and its barriers and motivators, adjusting for clustering effect and covariates using multiple logistic regression/multilevel models.

#### Qualitative data

For qualitative data, all the interviews will be transcribed verbatim in Bengali. Then we will review some interviews (e.g. 3–4 interviews) to develop initial code lists based on the interview guidelines and emerging themes for each group of respondents. After finalizing the code list, the team will code all the interviews and then summarise each code using the Dedoose software. Finally, we will conduct a thematic analysis using a framework method.

#### Data safety monitoring plan (DSMP)

The intervention involves awareness and ensuring the availability of vaccine supply. The information collected from the respondents will be kept private, using a unique identification number. Participants' data privacy will be ensured, and all data collected throughout the research will be kept strictly confidential and will not be shared with anyone else. Also, there will be strong controls on who has access to the data forms and rigorous adherence to maintaining the data confidentiality. There will be no sharing of participant names or any other identifying information with the information supplied by the survey respondents.

## Discussion

The current cluster randomized controlled trial protocol conducted in four of Bangladesh's tertiary level teaching hospitals will assess the effect of influenza vaccine awareness and the availability of vaccine supply intervention. Here, the quantitative approach will be used to estimate the effect of the intervention and elicit information on HCWs' knowledge and attitudes regarding influenza vaccination. The qualitative method will explore the barriers and motivators for influenza vaccine uptake and understand policymakers' views on the feasibility of influenza vaccination. The mixed methods design will strengthen the study findings by triangulating data collected from different approaches [45].

The WHO categorized HCWs as a priority risk group for influenza vaccination. Promoting influenza vaccination among HCWs would be important, particularly, for LMICs such as Bangladesh. The study will provide critical data on influenza vaccine uptake among Bangladeshi healthcare workers and the contextual and circumstantial factors influencing HCWs’ vaccine uptake. Firstly, the baseline data will inform the current practice regarding vaccination, including knowledge and perception about influenza illness and vaccination. Lessons from our interventions may help design context-specific vaccination campaigns and educational programs among HCWs to promote vaccine acceptability [46–48]. Secondly, increasing awareness regarding influenza vaccination among HCWs will likely improve adherence to receiving influenza vaccination annually. Thirdly, with improved knowledge on influenza vaccination and uptake, the HCWs can promote and advocate for influenza vaccine acceptance among other high-risk groups: elderly ≥ 65 years, adults with chronic diseases, pregnant women, and children six months to 5 years [24, 49].

The study results will not only generate critical data on vaccine uptake among HCWs but will also intend to capture data on HCWs’ and policy makers’ views on influenza vaccination among other high-risk populations to initiate further discussion on future immunization among different people at increased risk of influenza-like-illness.

## Conclusion

This intervention study will help fill the data gap on influenza vaccine uptake among HCWs in the LMIC healthcare setting. Major strengths of the protocol include an intervention study design and the utilization of qualitative methods. This study would generate valuable data on the programmatic feasibility of HCWs’ vaccination to observe the targeted influenza vaccination, HCWs’ vaccine uptake, and factors associated with uptake rates. Through this assessment, constraints faced for providing vaccines, the capacity of the health facility, preparedness, and the presence of logistical equipment for implementing the vaccination program will be articulated. Key findings from the study will be shared with the NITAG, which will be conveyed to the Ministry of Health for policy decisions regarding influenza vaccination among HCWs. This may help inform the MoH&FW on actions to be taken to increase awareness and develop an influenza vaccination policy.

## Supplementary Information

**Additional file 1:** **Supplementary Table 1.** Different types of hospitalhealthcare workers, Bangladesh.  

## Data Availability

Not applicable (For the purposes of this article, data sharing does not apply as no datasets have been produced or analyzed).

## References

[1] Guillari A, Polito F, Pucciarelli G, Serra N, Gargiulo G, Esposito MR, Botti S, Rea T, Simeone S: Influenza vaccination and healthcare workers: barriers and predisposing factors. A literature review. Acta Bio Medica: Atenei Parmensis 2021, 92(Suppl 2). 10.23750/abm.v92iS2.11106 PMC8138807 33855983

[2] Organization WH: Global influenza strategy 2019–2030. 2019.

[3] Iuliano AD, Roguski KM, Chang HH, Muscatello DJ, Palekar R, Tempia S, Cohen C, Gran JM, Schanzer D, Cowling BJ (2018). Estimates of global seasonal influenza-associated respiratory mortality: a modelling study. The Lancet.

[4] Tokars JI, Olsen SJ, Reed C (2018). Seasonal incidence of symptomatic influenza in the United States. Clin Infect Dis.

[5] Yassi A, McGill M, Holton D, Nicolle L (1993). Morbidity, cost and role of health care worker transmission in an influenza outbreak in a tertiary care hospital. Can J Infect Dis.

[6] Huttunen R, Syrjänen J (2014). Healthcare workers as vectors of infectious diseases. Eur J Clin Microbiol Infect Dis.

[7] Lietz J, Westermann C, Nienhaus A, Schablon A (2016). The occupational risk of influenza A (H1N1) infection among healthcare personnel during the 2009 pandemic: a systematic review and meta-analysis of observational studies. PLoS ONE.

[8] Kuster SP, Shah PS, Coleman BL, Lam P-P, Tong A, Wormsbecker A, McGeer A (2011). Incidence of influenza in healthy adults and healthcare workers: a systematic review and meta-analysis. PLoS ONE.

[9] Apisarnthanarak A, Puthavathana P, Kitphati R, Auewarakul P, Mundy LM (2008). Outbreaks of influenza A among nonvaccinated healthcare workers: implications for resource-limited settings. Infect Control Hosp Epidemiol.

[10] Somes MP, Turner RM, Dwyer LJ, Newall AT (2018). Estimating the annual attack rate of seasonal influenza among unvaccinated individuals: a systematic review and meta-analysis. Vaccine.

[11] Turnberg W, Daniell W, Duchin J (2010). Influenza vaccination and sick leave practices and perceptions reported by health care workers in ambulatory care settings. Am J Infect Control.

[12] Barberis I, Myles P, Ault S, Bragazzi N, Martini M (2016). History and evolution of influenza control through vaccination: from the first monovalent vaccine to universal vaccines. J Prev Med Hyg.

[13] Gasparini R, Amicizia D, Lai PL, Bragazzi NL, Panatto D (2014). Compounds with anti-influenza activity: present and future of strategies for the optimal treatment and management of influenza Part II: Future compounds against influenza virus. J Prev Med Hyg.

[14] Frenzel E, Chemaly RF, Ariza-Heredia E, Jiang Y, Shah DP, Thomas G, Graviss L, Raad I (2016). Association of increased influenza vaccination in health care workers with a reduction in nosocomial influenza infections in cancer patients. Am J Infect Control.

[15] Amodio E, Restivo V, Firenze A, Mammina C, Tramuto F, Vitale F (2014). Can influenza vaccination coverage among healthcare workers influence the risk of nosocomial influenza-like illness in hospitalized patients?. J Hosp Infect.

[16] Bénet T, Régis C, Voirin N, Robert O, Lina B, Cronenberger S, Comte B, Coppéré B, Vanhems P (2012). Influenza vaccination of healthcare workers in acute-care hospitals: a case-control study of its effect on hospital-acquired influenza among patients. BMC Infect Dis.

[17] Organization WH: Vaccines against influenza WHO position paper—November 2012. Weekly Epidemiological Record= Relevé épidémiologique hebdomadaire 2012, 87(47):461–476. 23210147

[18] mondiale de la Santé O, Organization WH: WHO seasonal influenza vaccination recommendations during the COVID-19 pandemic–Recommandations du SAGE de l’OMS pour la vaccination contre la grippe saisonnière pendant la pandémie de COVID-19. Weekly Epidemiological Record= Relevé épidémiologique hebdomadaire 2019, 95(45):539–543.

[19] mondiale de la Santé O, Organization WH: Vaccines against influenza: WHO position paper–May 2022–Vaccins antigrippaux: note de synthèse de l’OMS–mai 2022. Weekly Epidemiological Record= Relevé épidémiologique hebdomadaire 2022, 97(19):185–208.

[20] Al Awaidy S, Althaqafi A, Dbaibo G, East M, Network NAIS (2018). A snapshot of influenza surveillance, vaccine recommendations, and vaccine access, drivers, and barriers in selected Middle Eastern and North African Countries. Oman Med J.

[21] Hirve S, Organization WH: Seasonal influenza vaccine use in low and middle income countries in the tropics and subtropics: a systematic review: World Health Organization; 2015.

[22] Eaton JL, Mohr DC, McPhaul KM, Kaslow RA, Martinello RA: Access, education and policy awareness: predictors of influenza vaccine acceptance among VHA healthcare workers. infection control & hospital epidemiology 2017, 38(8):970–975. 10.1017/ice.2017.113 28641586

[23] Abu-Gharbieh E, Fahmy S, Rasool BA, Khan S (2010). Influenza vaccination: healthcare workers attitude in three Middle East countries. Int J Med Sci.

[24] Rashid H, Yin JK, Ward K, King C, Seale H, Booy R (2016). Assessing interventions to improve influenza vaccine uptake among health care workers. Health Aff.

[25] Looijmans-Van Den Akker I, Van Delden J, Verheij TJ, van der Sande M, Van Essen G, Riphagen-Dalhuisen J, Hulscher M, Hak E: Effects of a multi-faceted program to increase influenza vaccine uptake among health care workers in nursing homes: A cluster randomised controlled trial. Vaccine 2010, 28(31):5086–5092. 10.1016/j.vaccine.2010.05.003 20580740

[26] Dini G, Toletone A, Sticchi L, Orsi A, Bragazzi NL, Durando P (2018). Influenza vaccination in healthcare workers: A comprehensive critical appraisal of the literature. Hum Vaccin Immunother.

[27] Gurley ES, Zaman RU, Sultana R, Bell M, Fry AM, Srinivasan A, Rahman M, Rahman MW, Hossain MJ, Luby SP (2010). Rates of hospital-acquired respiratory illness in Bangladeshi tertiary care hospitals: results from a low-cost pilot surveillance strategy. Clin Infect Dis.

[28] Hassan MZ, Sturm-Ramirez K, Rahman MZ, Hossain K, Aleem MA, Bhuiyan MU, Islam MM, Rahman M, Gurley ES (2019). Contamination of hospital surfaces with respiratory pathogens in Bangladesh. PLoS ONE.

[29] Ip DK, Lau EH, Tam YH, So HC, Cowling BJ, Kwok HK (2015). Increases in absenteeism among health care workers in Hong Kong during influenza epidemics, 2004–2009. BMC Infect Dis.

[30] Hirve S, Lambach P, Paget J, Vandemaele K, Fitzner J, Zhang W (2016). Seasonal influenza vaccine policy, use and effectiveness in the tropics and subtropics–a systematic literature review. Influenza Other Respir Viruses.

[31] Saudi Arabia makes vaccine mandatory for hajj pilgrims. The Daily Star. https://www.thedailystar.net/news-detail-113277. Accessed March 22 2022

[32] List of Primary, 2ndary & Tertiary Level Hospital. Hospital & Clinic Section, Director General of Health Service, Ministry ofHealth and Social Welfare, Bangaldesh. http://hospitaldghs.gov.bd/list-of-2ndary-tertiary-level-hospital/. Accessed March 22 2022. In.

[33] mondiale de la Santé O, Organization WH: Recommended composition of influenza virus vaccines for use in the 2022–2023 northern hemisphere influenza season–Composition recommandée des vaccins antigrippaux pour la saison grippale 2022–2023 dans l’hémisphère Nord. Weekly Epidemiological Record= Relevé épidémiologique hebdomadaire 2022, 97(12):109–119.

[34] Zaman RU, Alamgir A, Rahman M, Azziz-Baumgartner E, Gurley ES, Sharker MAY, Brooks WA, Azim T, Fry AM, Lindstrom S (2009). Influenza in outpatient ILI case-patients in national hospital-based surveillance, Bangladesh, 2007–2008. PLoS ONE.

[35] Cox N: Influenza seasonality: timing and formulation of vaccines. In., vol. 92: SciELO Public Health; 2014: 311–311. 10.2471/BLT.14.139428 PMC4007136 24839317

[36] Akhtar Z, Chowdhury F, Rahman M, Ghosh PK, Ahmmed MK, Islam MA, Mott JA, Davis W (2021). Seasonal influenza during the COVID-19 pandemic in Bangladesh. PLoS ONE.

[37] Seasonal Influenza Vaccination Resources for Health Professionals. CDC .https://www.cdc.gov/flu/professionals/vaccination/index.htm. Accessed March 23 2022.

[38] Information for Health Professionals. CDC .https://www.cdc.gov/flu/professionals/index.htm. Accessed March 23 2022.

[39] Organization WH: How to implement seasonal influenza vaccination of health workers: an introduction manual for national immunization programme managers and policy makers: pilot version 2019. 2019.

[40] Madewell ZJ, Chacón-Fuentes R, Jara J, Mejía-Santos H, Molina I-B, Alvis-Estrada JP, Ortiz M-R, Coello-Licona R, Montejo B (2021). Knowledge, attitudes, and practices of seasonal influenza vaccination in healthcare workers, Honduras. PLoS ONE.

[41] Hammour WA, Al-Saleh S (2019). Knowledge, attitudes, and practice of healthcare workers toward influenza vaccination at Al Jalila Children's Specialty Hospital (AJCH), Dubai, UAE. International Journal of Pediatrics and Adolescent Medicine.

[42] Alshammari TM, Yusuff KB, Aziz MM, Subaie GM (2019). Healthcare professionals’ knowledge, attitude and acceptance of influenza vaccination in Saudi Arabia: a multicenter cross-sectional study. BMC Health Serv Res.

[43] Oguz MM: Improving influenza vaccination uptake among healthcare workers by on-site influenza vaccination campaign in a tertiary children hospital. Human vaccines & immunotherapeutics 2019. 10.1080/21645515.2019.1575164 PMC6605858 30735439

[44] Cherif I, Kharroubi G, Bouabid L, Gharbi A, Boukthir A, Ben Alaya N, Ben Salah A, Bettaieb J (2021). Knowledge, attitudes and uptake related to influenza vaccine among healthcare workers during the 2018–2019 influenza season in Tunisia. BMC Public Health.

[45] Schoonenboom J, Johnson RB (2017). How to construct a mixed methods research design. KZfSS Kölner Zeitschrift für Soziologie und Sozialpsychologie.

[46] Naleway AL, Henkle EM, Ball S, Bozeman S, Gaglani MJ, Kennedy ED, Thompson MG (2014). Barriers and facilitators to influenza vaccination and vaccine coverage in a cohort of health care personnel. Am J Infect Control.

[47] Petek D, Kamnik-Jug K (2018). Motivators and barriers to vaccination of health professionals against seasonal influenza in primary healthcare. BMC Health Serv Res.

[48] Alame M, Kaddoura M, Kharroubi S, Ezzeddine F, Hassan G, Diab El-Harakeh M, Al Ariqi L, Abubaker A, Zaraket H (2021). Uptake rates, knowledge, attitudes, and practices toward seasonal influenza vaccination among healthcare workers in Lebanon. Hum Vaccin Immunother.

[49] Costantino C, Restivo V, Gaglio V, Lanza G, Marotta C, Maida C, Mazzucco W, Casuccio A, Torregrossa M, Vitale F (2019). Effectiveness of an educational intervention on seasonal influenza vaccination campaign adherence among healthcare workers of the Palermo University Hospital. Italy Ann Ig.

