# Supplementary material for: Seasonal influenza vaccine uptake among healthcare workers in tertiary care hospitals, Bangladesh: Study protocol for influenza vaccine supply and awareness intervention
Source: BMC Public Health. 2022 Sep 24;22:1819. doi: 10.1186/s12889-022-14182-w (PMC9509585; doi:10.1186/s12889-022-14182-w)
Supplement: Supplementary file 1 — Additional file 1: Supplementary Table 1. Different types of hospitalhealthcare workers, Bangladesh. [file 12889_2022_14182_MOESM1_ESM.docx]

**Supplementary Table 1: Different types of hospital healthcare workers, Bangladesh**

| Healthcare worker type | Designation |
| --- | --- |
| Physician/ Doctor | Director, Deputy Director, Deputy superintendent, Assistant Director, Indoor Medical Officer, Resident Surgeon, Assistant Surgeon, Assistant, dental surgeon, Radiologist, Registrar, Assistant Registrar, Assistant Radiologist, Consultant, Senior Consultant, Junior Consultant, Medical officer (MO), Resident Medical Officer (RMO), Resident Physician, Emergency Medical Officer, Resident Psychiatrist, Anesthetist, Clinical Pathologist, Clinical Psychiatrist, Medical Physicist, , Clinical Microbiologist, House Surgeon, Neurologist, Dental surgeon, Intern, Postgraduate trainee |
| Nurse | Deputy Metron, Deputy Nursing Superintendent, Senior Metron, Junior Metron, Metron, Midwife, Nursing Supervisor, Senior staff nurse, Staff nurse, Intern nurse |
| Allied health personnel | Medical Technologist, Anesthesia Technician, Angiogram Technician, Attendant, Auto Clave Operator, Aya, Blood Bank Attendant, Cleaner, Clerk, Cold Chain worker, Counselor, OT Boy |
